# D-Amino Acids in the Nervous and Endocrine Systems

**DOI:** 10.1155/2016/6494621

**Published:** 2016-12-08

**Authors:** Yoshimitsu Kiriyama, Hiromi Nochi

**Affiliations:** Kagawa School of Pharmaceutical Sciences, Tokushima Bunri University, Shido 1314-1, Sanuki, Kagawa 769-2193, Japan

## Abstract

Amino acids are important components for peptides and proteins and act as signal transmitters. Only L-amino acids have been considered necessary in mammals, including humans. However, diverse D-amino acids, such as D-serine, D-aspartate, D-alanine, and D-cysteine, are found in mammals. Physiological roles of these D-amino acids not only in the nervous system but also in the endocrine system are being gradually revealed. N-Methyl-D-aspartate (NMDA) receptors are associated with learning and memory. D-Serine, D-aspartate, and D-alanine can all bind to NMDA receptors. H_2_S generated from D-cysteine reduces disulfide bonds in receptors and potentiates their activity. Aberrant receptor activity is related to diseases of the central nervous system (CNS), such as Alzheimer's disease, amyotrophic lateral sclerosis, and schizophrenia. Furthermore, D-amino acids are detected in parts of the endocrine system, such as the pineal gland, hypothalamus, pituitary gland, pancreas, adrenal gland, and testis. D-Aspartate is being investigated for the regulation of hormone release from various endocrine organs. Here we focused on recent findings regarding the synthesis and physiological functions of D-amino acids in the nervous and endocrine systems.

## 1. Introduction

Amino acids are important not only as essential components for the building blocks of peptides and proteins but also as biochemical regulators, such as neurotransmitters [1–4] and autophagy regulators [5–8]. D-Amino acids are enantiomers of L-amino acids and have been considered to be absent and unnatural amino acids in mammals for a long time. However, the recent development of sensitive analytical methods elucidated the presence of D-amino acids, such as D-serine, D-aspartate, and D-alanine, in mammals [9–11]. Moreover, studies on the enzymes that synthesize or metabolize D-amino acids have also clarified the localization and functions of D-amino acids in the nervous and endocrine systems and found that D-amino acid synthesis and metabolism are physiologically regulated [12–15]. Here we focused on recent advances in understanding the synthesis, metabolism, and physiological roles of D-amino acids in the nervous and endocrine systems.

## 2. D-Serine

Marked levels of D-serine were found in the central nervous system (CNS) of rodents and humans. D-Serine-abundant regions in the CNS were the cerebral cortex, hippocampus, and striatum. Additionally, D-serine is also detectable in other regions, such as the midbrain, cerebellum, and spinal cord, of rodents and humans [16–19]. The extracellular levels of D-serine in the medial prefrontal cortex and striatum of rats are approximately 20% of the total level of serine [20]. D-Serine is biosynthesized by serine racemase (SR) in the CNS of rodents and humans [21–23]. In addition, the level of D-serine in the CNS is considerably decreased in* Sr* knockout mice [24, 25].

**Figure 1:**
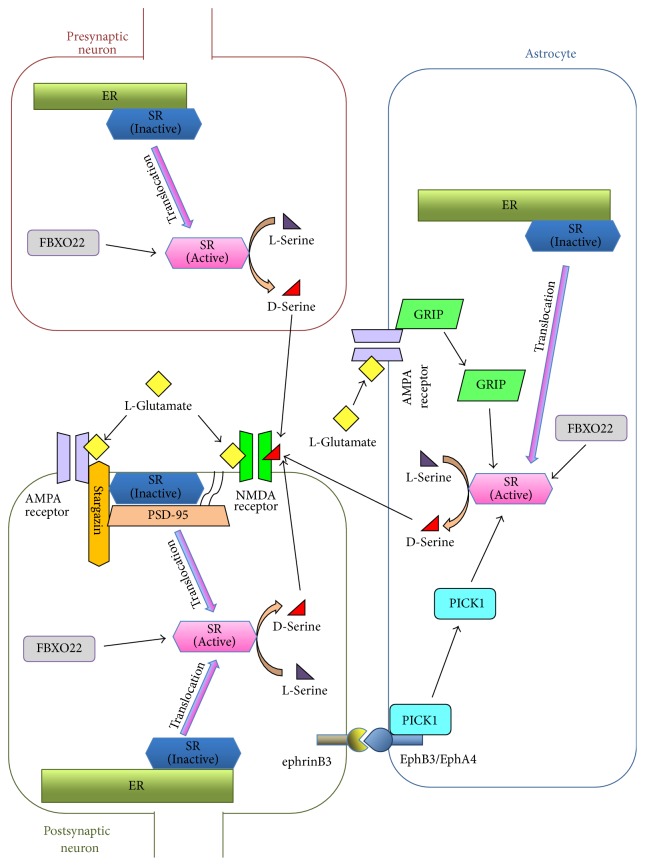
The synthesis of D-serine in the central nervous systems (CNS). D-Serine is synthesized by serine racemase (SR). SR is inhibited by its translocation from the cytosol to a membrane, such as the endoplasmic reticulum (ER) or plasma membranes, all of which contain phosphatidylinositol 4,5-bisphosphate (PIP_2_). F-box only protein 22 (FBXO22) interacts with SR and activates SR by preventing it from binding to the ER membrane. In astrocytes, glutamate receptor interacting protein 1 (GRIP1) binds to the *α*-amino-3-hydroxy-5-methylisoxazole-4-propionic acid (AMPA) receptor and GRIP1 is then released from the AMPA receptor following stimulation with L-glutamate. Released GRIP1 activates SR. Protein interacting with C-kinase (PICK1) binds to erythropoietin-producing hepatocellular receptor (Eph)B3 or EphA4 in the astrocytes. PICK1 is released after ephrinB3 on the neurons interacts with the EphB3 or EphA4 receptor and then activates SR. Stargazin forms a complex with the AMPA receptor, postsynaptic density protein 95 (PSD-95), and SR and inhibits the activity of SR by promoting membrane localization in neurons. After the AMPA receptor is activated, SR is released from the plasma membrane, resulting in the activation of SR. The N-methyl-D-aspartate (NMDA) receptor is activated by glutamate and D-serine.

It was shown that SR localizes to astrocytes as well as neurons and D-serine was released from both [22, 23, 26–29]. In addition, D-serine in the neurons is generated from L-serine provided from astrocytes [27]. The D-serine shuttle model describes optimal D-serine-mediated N-methyl-D-aspartate (NMDA) receptor activity by proposing that D-serine is transported between neurons and astrocytes [30, 31]. This D-serine shuttle model is as follows. Astrocytes uptake glucose from the blood vessels via glucose transporter 1 and then 3-phosphoglycerate dehydrogenase (Phgdh), which is mainly localized in astrocytes and converts glucose to L-serine. L-Serine is exported from astrocytes and imported into neurons through alanine/serine/cysteine/threonine transporters (ASCTs). In the neuron, L-serine is converted to D-serine by SR. D-serine is released from neurons, through alanine-serine-cysteine transporter-1 (Asc-1) or other pathways, into the synapse where it can regulate NMDA receptor activity. Released D-serine can also be imported into astrocytes through ASCTs. The ability of SR to racemize L-serine to D-serine is positively regulated by pyridoxal-5′-phosphate (PLP), divalent cations, and ATP [22, 32, 33]. On the other hand, the activity of SR is inhibited by its translocation from the cytosol to membranes that contain phosphatidylinositol 4,5-bisphosphate (PIP_2_), such as the nuclear, endoplasmic reticulum (ER), and plasma membranes [34–37]. SR is also regulated by interaction with other proteins (Figure 1). Glutamate receptor interacting protein 1 (GRIP1) [38] and protein interacting with C-kinase (PICK1) [39, 40] have been found to activate SR by interacting with it in the astrocytes of rodents. GRIP1 binds to the *α*-amino-3-hydroxy-5-methylisoxazole-4-propionic acid (AMPA) receptor and is released following stimulation with L-glutamate. The released GRIP1 binds and activates SR [38]. The postsynaptic density protein 95 (PSD95)/Discs large/ZO-1 (PDZ) domain in the C-terminal region of GRIP1 was found to be responsible for the interaction with SR [38, 41]. PICK1 has a PDZ domain that binds to SR [39] and a Bin/amphiphysin/Rvs (BAR) domain that interacts with membranes [42]. PICK1 associates with erythropoietin-producing hepatocellular receptor (Eph)B3 or EphA4 in astrocytes. After ephrinB3 that is expressed on postsynaptic neurons binds to the EphB3 or EphA4 receptor, PICK1 is released into the cytosol. Enhancement of the association between the released PICK1 and SR increases the synthesis of D-serine in the astrocytes of mice [40]. Stargazin and F-box only protein 22 (FBXO22) also regulate SR activity by affecting subcellular localization of SR. Stargazin forms a complex with the resting AMPA receptor, PSD-95, and SR, inhibiting the activity of SR by promoting membrane localization in the neurons of mice. Once the AMPA receptor is activated, SR is released from stargazin and the plasma membrane, leading to the activation of SR [43]. FBXO22 binds and activates SR by preventing its binding to the ER membrane in the neurons and astrocytes of rats. FBXO22 may block the lipid-binding region of SR [35].

D-Serine is catalyzed by SR and D-amino acid oxidase (DAO). SR has *α*,*β*-elimination and racemization activities. SR converts both D-serine and L-serine to generate pyruvate and ammonia by removing water from these amino acids by *α*,*β*-elimination [44]. Therefore, SR might regulate the physiological level of D-serine by racemization activity to synthesize D-serine and by *α*,*β*-elimination activity to degrade D-serine. DAO is the flavoprotein that converts D-serine to produce the corresponding alpha keto acid, hydrogen peroxide, and ammonia [11].

The distribution of D-serine in the CNS was found to be similar to that of NMDA receptors, which are ionotropic glutamate receptors (iGluRs) [18, 45], and the activation of NMDA receptors requires the binding of both glutamate and D-serine or glycine. The activated NMDA receptors induce Ca^2+^ flow, leading to the regulation of long-term potentiation (LTP) and long-term depression (LTD) in different areas of the CNS. Thus, the regulation of NMDA receptors is highly associated with synaptic activities, learning, and memory [46–49].

The NMDA receptor is mainly composed of two GluN1 subunits and two GluN2 subunits of either same or different GluN2 subunits. Glutamate binds to GluN2, whereas D-serine or glycine binds to GluN1 [50–52]. Although D-serine or glycine can act as the coagonist for NMDA receptors [46], reduction in the level of D-serine diminishes the NMDA receptor activity and addition of D-serine reverses this inactivation, leading to LTP [46, 53]. Moreover, D-serine induces LTP, and the degradation of D-serine by DAO leads to the suppression of this induction [49]. Therefore, D-serine plays important roles in the regulation of synaptic activities, learning, and memory by regulating the activation of NMDA receptors. However, neurotoxic effects of D-serine have been reported. Compared with wild-type mice, reduced levels of D-serine in adult* Sr* knockout mice diminished NMDA receptor-mediated and *β*-amyloid_1–42_-induced neurotoxicity [24]. Moreover, elevated levels of D-serine in adult* Dao* knockout mice resulted in motor neuron degeneration [54]. Aberrant levels of D-serine are associated with diseases caused by abnormal NMDA receptor activity. Levels of D-serine in the cerebrospinal fluid (CSF) of patients with Alzheimer's disease were reported to be higher than those reported in normal controls [55, 56]. In addition, beta-amyloid-induced neurotoxicity is suppressed in* Sr* knockout mice, which showed a 90% decrease in the level of D-serine in the brain [24]. Schizophrenia is associated with hypofunction of NMDA receptors [57]. Decreased D-serine level resulted in hypofunction of NMDA receptors and this leads to schizophrenia-like symptoms [25, 58]. Moreover, administration of D-serine ameliorates positive, negative, and cognitive symptoms in patients with schizophrenia [59]. Amyotrophic lateral sclerosis (ALS) is related with increased levels of D-serine [60, 61]. Therefore, controlling the level of D-serine might be one of therapeutic targets for these diseases.

On the other hand, D-serine binds to the *δ*2-type glutamate receptor (GluD2), which is iGluR, and drives the reduction of AMPA receptors in Purkinje cells in the cerebellum by endocytosis. This results in the promotion of cerebellar LTD. Moreover, binding of D-serine to GluD2 leads to the acquisition of motor learning in mice [62]. Recently, it has been reported that the age-dependent increase in pyruvate carboxylase activity in glial cells causes a decrease in the level of D-serine, leading to age-related memory impairment in flies [63]. Pyruvate carboxylase generates oxaloacetic acid, which can be converted to aspartic acid. Both oxaloacetic acid and aspartic acid can inhibit SR, resulting in a decrease in the levels of D-serine [64].

D-Serine has also been found in endocrine organs, such as the adrenal and pituitary glands, pancreas, and testis, of rats [65, 66]. However, levels of D-serine in the endocrine system are much lower than those in the CNS, and the physiological role of D-serine in the endocrine systems remains unclear.

## 3. D-Aspartate

D-Aspartate is found in the CNS of rodents and humans [16, 19, 67–72] as well as in endocrine organs, including the pineal gland [69, 72, 73], pituitary gland [68, 69, 71, 72, 74], pancreas [66], adrenal gland [19, 67, 72, 74, 75], and testis, of rats [74, 76]. D-Aspartate activates NMDA receptors via binding to the agonist site of GluN2 subunits (2A–D) [77–79] and can also activate metabotropic glutamate receptor 5 (mGlu5) [80]. Although the enzyme that generates D-aspartate has not been identified yet [81–83], the generation of D-aspartate might be dependent on PLP [67].

On the other hand, it has been demonstrated that D-aspartate is degraded to oxaloacetate, hydrogen peroxide, and ammonium by the peroxisomal flavoprotein D-aspartate oxidase (DDO) [84–86]. Although D-aspartate is abundant in the brains of rodents and humans during development, levels of D-aspartate are extremely decreased at postnatal stages and this reduction remains throughout adulthood [16, 67, 68, 71, 87, 88]. In contrast, DDO activity and its mRNA are exceedingly low at postnatal stages and increase after birth in rodents [88, 89]. Moreover, it has been shown that D-aspartate levels were significantly elevated in the CNS of* Ddo* knockout mice [88, 90–93]. In addition, D-aspartate is found exclusively in the neurons of rats [67]. Improvement in spatial memory was demonstrated in* Ddo* knockout mice [77, 78]. Moreover, D-aspartate-treated mice and* Ddo* knockout mice demonstrated an enhancement of LTP [77–79] and increase in the dendritic length and spine density in neurons in the prefrontal cortex and hippocampus [94]. Therefore, a significant increase in levels of D-aspartate, enhancement of LTP, and increase in the dendritic length and spine density of neurons in adult* Ddo* knockout mice indicate that D-aspartate might also be generated and function in CNS during adulthood. However, persistent elevation in levels of D-aspartate in adult* Ddo* knockout mice has neurotoxic effects, such as activation of caspase-3 and apoptosis in neurons [88]. The expression of* Ddo* mRNA in postmortem brains of patients with schizophrenia was significantly higher than that in control individuals [95]. Moreover, it has been reported that the levels of D-aspartate were significantly reduced in the postmortem brains of patients with schizophrenia compared with those in control individuals [96].* Ddo* knockout mice exhibit reduced schizophrenia-like behaviors induced by phencyclidine, such as motor hyperactivity and prepulse inhibition [95]. These findings indicate that an aberrant level of D-aspartate may be associated with schizophrenia.

In the endocrine system, D-aspartate controls the synthesis and release of hormones. D-Aspartate inhibits the synthesis of melatonin in the rat pineal gland [97] and also reduces the release of melatonin from cultured rat pinealocytes [98]. In the rat hypothalamus, D-aspartate induces oxytocin and vasopressin gene expression [99] and may increase the release of gonadotropin-releasing hormone [100]. D-Aspartate also induces the release of prolactin [101], growth hormone, and luteinizing hormone [100, 102] from the rat anterior pituitary. In contrast, an increase in the levels of D-aspartate is associated with considerable decrease in proopiomelanocortin and *α*-melanocyte-stimulating hormone in the mouse intermediate lobe [93]. In the rat testis, D-aspartate induces the synthesis and release of testosterone [102–104]. Upregulated androgen and downregulated estrogen receptor expressions by D-aspartate administration were shown in rat testis [104]. Moreover, the oral intake of D-aspartate induces the release of testosterone in human serum [102] and ameliorates the number and motility of the spermatozoa in humans [105]. Therefore, it is expected that D-aspartate could be a candidate for infertility treatment.

## 4. Other D-Amino Acids

Various D-amino acids other than D-serine and D-aspartate were also found in the nervous and endocrine systems.

D-Alanine is detected in the brain, pituitary gland, pancreas, adrenal gland, and testis of rodents [106]. In addition, it is also detected in the human brain [107]. Most of the D-alanine in rodents is derived from intestinal bacteria [108, 109]. D-Alanine is metabolized by DAO [11], and the levels of D-alanine in rodents depend on the circadian rhythm [9, 110]. In addition, it has been reported that D-alanine binds to the NMDA receptor and improves the symptoms of patients with schizophrenia [111].

**Figure 2:**
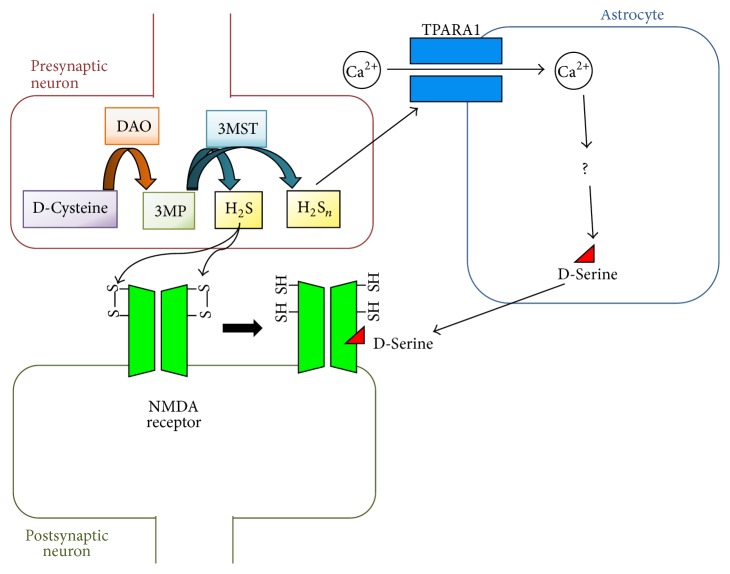
Activation of the N-methyl-D-aspartate (NMDA) receptor by D-cysteine. D-Cysteine is one of the sources of H_2_S in the brain. D-Cysteine is converted to 3-mercaptopyruvate (3MP) by D-amino acid oxidase (DAO). 3MP is then degraded by 3-mercaptopyruvate sulfurtransferase (3MST) to generate H_2_S in the neurons. Hydrogen polysulfides (H_2_S_*n*_; *n* = 2–5) are also generated by 3MST from 3MP. H_2_S reduces the disulfide bonds in the NMDA receptor and increases the activity of the NMDA receptor. H_2_S_*n*_ also activates the transient receptor potential (TRP) A1 channel. Activated TRPA1 channel induces Ca^2+^ influx, leading to D-serine release in astrocytes, and D-serine then activates the NMDA receptor.

D-Cysteine was found to be one of sources of H_2_S in the brain (Figure 2). D-Cysteine is probably absorbed at least partially from food, although the source of D-cysteine in the body has not been elucidated thus far [112]. In the CNS, D-cysteine is degraded by DAO to generate 3-mercaptopyruvate (3MP). 3MP is then catalyzed by 3-mercaptopyruvate sulfurtransferase (3MST) to produce H_2_S [113]. 3MST is found in the synaptosomes and neurons of mouse brains [114]. Moreover, hydrogen polysulfides (H_2_S_*n*_; *n* = 2–5) are found in the mouse brain and are also generated by 3MST from 3MP [115]. H_2_S enhances the activity of NMDA receptors by reducing disulfide bonds in NMDA receptors and induces LTP in rats [116, 117]. In contrast, H_2_S_*n*_ activates the transient receptor potential (TRP) A1 channels in rodent astrocytes and induces the flow of Ca^2+^ [118]. Activation of TRPA1 channels leads to D-serine release from rodent astrocytes and enhances NMDA receptor-dependent LTP [119]. In addition, Parkin is one of the key factors for Parkinson's disease [7], and H_2_S enhances the activity of Parkin and leads to protective effects against Parkinson's disease [120]. In the endocrine system, H_2_S inhibits glucose-induced insulin release from pancreatic *β* cells [121].

D-leucine and D-proline are found in the brain and pineal and pituitary gland of rodents [122, 123]. D-glutamate is found in the rodent brain [70, 124]. However, detailed physiological roles of these amino acids remain unclear.

## 5. Conclusions

It has been considered that only L-amino acids are utilized in mammals, including humans. However, because of the recent development of sensitive and selective analytical methods for detecting chiral amino acids [125], diverse D-amino acids have been found in mammalian tissues. The physiological functions of these D-amino acids are being gradually clarified. It has been demonstrated that D-amino acids, such as D-serine, D-aspartate, D-alanine, and D-cysteine, play important roles in the nervous and endocrine systems. Therefore, it is very important that the mechanisms of synthesis and metabolism as well as the physiological functions of D-amino acids are investigated further. These investigations will provide new therapeutic and diagnostic strategies for diseases related to the nervous and endocrine systems.
